# A Multicenter Prospective Study to Investigate the Diagnostic Accuracy of the SeHCAT Test in Measuring Bile Acid Malabsorption: Research Protocol

**DOI:** 10.2196/resprot.4467

**Published:** 2016-02-01

**Authors:** Fiona Reid, Janet Peacock, Bola Coker, Viktoria McMillan, Cornelius Lewis, Stephen Keevil, Roy Sherwood, Gill Vivian, Robert Logan, Jennifer Summers

**Affiliations:** ^1^ Division of Health and Social Care Research King's College London London United Kingdom; ^2^ NIHR Biomedical Research Centre at Guy’s and St Thomas’ NHS Foundation Trust and King's College London London United Kingdom; ^3^ King's Technology Evaluation Centre (KiTEC) King's College London London United Kingdom; ^4^ Division of Imaging Sciences & Biomedical Engineering King's College London London United Kingdom; ^5^ Department of Medical Engineering and Physics King’s College Hospital NHS Foundation Trust London United Kingdom; ^6^ Department of Medical Physics Guy’s and St Thomas’ NHS Foundation Trust London United Kingdom; ^7^ Department of Clinical Biochemistry Viapath King’s College Hospital NHS Foundation Trust London United Kingdom; ^8^ Department of Nuclear Medicine King’s College Hospital NHS Foundation Trust London United Kingdom; ^9^ Department of Gastroenterology King’s College Hospital NHS Foundation Trust London United Kingdom

**Keywords:** diagnostic test, accuracy, bile acid malabsorption, diarrhea, SeHCAT test, bile acid sequestrant

## Abstract

**Background:**

Bile acid malabsorption (BAM) is one possible explanation for chronic diarrhea. BAM may be idiopathic, or result from ileal resection or inflammation including Crohn’s disease, or may be secondary to other conditions, including cholecystectomy, peptic ulcer surgery, and chronic pancreatitis. No “gold standard” exists for clinical diagnosis of BAM, but response to treatment with a bile acid sequestrant (BAS) is often accepted as confirmation. The SeHCAT (tauroselcholic [selenium-75] acid) test uses a radiolabeled synthetic bile acid and provides a diagnostic test for BAM, but its performance against “trial of treatment” is unknown. Fibroblast growth factor 19 (FGF-19) and 7-alpha-hydroxy-4-cholesten-3-one (C4) also offer potential new biomarkers of BAM.

**Objective:**

This protocol describes a multicenter prospective study to evaluate the diagnostic accuracy of SeHCAT and 2 biomarkers in predicting BAM as assessed by trial of treatment.

**Methods:**

Participating gastroenterology centers should have a minimum workload of 30 SeHCAT patients per annum. Patients should not be pregnant, on medication that could confound follow-up, or have any severe comorbidity. All eligible patients attending a gastrointestinal appointment will be invited to participate. On attending the SeHCAT test, blood and fecal samples will be collected for analysis of FGF-19 by enzyme-linked immunosorbent assay and for C4 and fractionated bile acids by liquid chromatography–mass spectrometry. A capsule containing radiolabeled SeHCAT will be administered orally and a scan performed to measure SeHCAT activity. Patients will return on day 7 to undergo a second scan to measure percentage SeHCAT retention. The test result will be concealed from clinicians and patients. BAS will be dispensed to all patients, with a follow-up gastroenterologist appointment at 2 weeks for clinical assessment of treatment response and adherence. Patients responding positively will continue treatment for a further 2 weeks and all patients will have a final follow-up at 8 weeks. The diagnostic accuracy of the SeHCAT test and biomarkers will be analyzed at different thresholds using sensitivity, specificity, positive and negative predictive value, likelihood ratios, and area under the curve in a sample of 600 patients. Multivariable logistic regression models will be used to assess the association between presence of BAM and continuous SeHCAT retention levels after adjustment for confounders.

**Results:**

Funding is being sought to conduct this research.

**Conclusions:**

The SeHCAT test for diagnosis of BAM has been in common use in the United Kingdom for more than 30 years and an evidence-based assessment of its accuracy is overdue. The proposed study has some challenges. Some forms of BAS treatment are unpleasant due to the texture and taste of the resin powder, which may negatively affect recruitment and treatment adherence. Trial of treatment is not as “golden” a standard as would be ideal, and itself warrants further study.

## Introduction

Chronic diarrhea is a common problem and the investigation and management of the condition places a significant burden on health services as well as on affected patients. The suggestion that bile acid malabsorption (BAM) can cause diarrhea was first described by Hofmann in 1967 [1]; since then, BAM has been identified as a possible explanation for persistent chronic diarrhea.

Bile acids are produced in the liver, stored in the gallbladder, and released upon eating for the digestion of dietary fat. They are then largely reabsorbed by the terminal ileum and returned to the liver, a process known as enterohepatic circulation. When the reabsorption process is disrupted, excess levels of bile acids enter the colon where they lead to increased motility and water secretion, resulting in diarrhea.

Evidence is accumulating that BAM is more common than was previously thought [2,3]; however, robust data on the prevalence of BAM do not exist. Three types of BAM have been defined. In BAM Type 1, the bile acid malabsorption results from ileal resection, ileal disease including Crohn’s disease, or bypass of the terminal ileum. BAM Type 2 relates to primary, idiopathic malabsorption, whereas BAM Type 3 represents malabsorption secondary to other conditions, including cholecystectomy, peptic ulcer surgery, chronic pancreatitis, and celiac disease.

A diagnostic test for BAM is provided by SeHCAT (tauroselcholic [selenium-75] acid), a radiolabeled synthetic bile acid. The SeHCAT test is a measure of the retention of radioactivity in the patient following administration of a capsule containing SeHCAT. The patient is scanned with a gamma camera 1 to 3 hours after taking the capsule and the scanning is repeated after 7 days to measure the percentage retention of the radiolabeled bile acid. A low SeHCAT retention level at day 7 represents an abnormal result for the test, indicating a positive diagnosis of BAM.

The SeHCAT test was introduced in the late 1970s; however, despite being used for more than 30 years, much of the available evidence is anecdotal knowledge built up over time rather than through systematic research. A recent survey on the use of SeHCAT in the United Kingdom provided an insight into the frequency of use of the test and the practicalities of its implementation in UK hospitals [4]. The study identified 73 centers using SeHCAT with a wide variation in the annual patient workload ranging from 1 to 300 tests (mean 51; median 30). An increase in referrals since 2010 was reported in response to demand from clinicians. Considerable variability in practical implementation of the technique was found, alongside a wide variation in the “normal” range of the SeHCAT percentage retention levels used for reporting, diagnosis, and treatment. The different approaches to definition of an abnormal result included a single threshold value (of which <15% retention was the most common), division into 3 categories (normal, borderline, and abnormal), or into 4 categories (normal, mild, moderate, and severe).

Patients with a diagnosis of BAM from a positive SeHCAT test may be offered treatment with bile acid sequestrants (BAS), such as cholestyramine, colestipol, and colesevelam. In general, patients who adhere to BAS treatment respond well and rapidly (within days) with significant reduction in bowel frequency and improvements in their quality of life [3]. However, certain BAS treatments are unpleasant to the patient and clear communication between the clinician and the patient is needed to highlight the expected benefits of treatment to ensure good adherence. Studies have found a relationship between SeHCAT retention levels and response to BAS treatment, with lower retention levels associated with greater resolution of symptoms [5].

A prospective survey was conducted in 2014 by the King’s Technology Evaluation Centre (KiTEC) to characterize the clinical indications for referring patients for a SeHCAT test across 38 UK centers and to describe the range of test results and treatment pathways [6]. Patients with BAM Type 1 represented 14% of more than 700 patients tested with the remainder split fairly equally between BAM Types 2 and 3. Using center-defined thresholds, 51% of results were defined as abnormal or borderline; however, only 37% of patients were prescribed treatment with BAS. Median SeHCAT retention levels were much lower for BAM Type 1 (2%) compared with BAM Types 2 and 3 (18% and 17%, respectively).

The diagnostic performance of the SeHCAT test across the range of thresholds in current use is poorly understood. There is no established “gold standard” for the diagnosis of BAM. However, diagnosis is sometimes made through a trial of treatment with BAS and response to treatment with BAS offers a potential reference standard, if taken by *all* patients, not just those who the SeHCAT test suggests may be in the abnormal range. A review by Riemsma et al [7] identified 3 studies which had taken this approach; however, the numbers were small (ranging from 13 to 46 patients) resulting in wide confidence intervals [7-10]. Specificity was more than 0.9 in all 3 studies; in the 2 studies that used a cut-off of 8% retention, sensitivity ranged from 0.67 to 0.95. Further research using larger samples is needed to assess the accuracy of the SeHCAT test in diagnosing the BAM condition as determined by response to BAS treatment.

In addition, 2 biomarkers have shown promising results as possible predictors of BAM, namely fibroblast growth factor 19 (FGF-19) and 7-alpha-hydroxy-4-cholesten-3-one (C4) [11,12]. Exploration of the performance of these biomarkers as alternative tools for discriminating between diagnoses could potentially improve diagnostic accuracy.

This protocol describes a study that will assess the diagnostic performance of the SeHCAT test and of 2 biomarkers in the prediction of BAM using a positive response to treatment with BAS as a definitive indication of the BAM condition. Improved diagnostic accuracy for BAM should, in turn, allow treatment of this debilitating condition to be optimized.

### Objectives

The primary research objective is to investigate the diagnostic accuracy of the SeHCAT test in providing a positive indication of BAM among people with chronic diarrhea who are clinically suspected of having BAM, using trial of treatment with BAS as the gold standard.

The secondary research objectives are to investigate:

1. The continuum of results of the SeHCAT test, overall and for different clinical populations;
2. The feasibility of using the SeHCAT test to provide an indication of the severity of BAM (eg, mild, moderate, or severe);
3. Adherence to treatment with BAS, overall and for different clinical populations;
4. The diagnostic accuracy of 2 biomarker tests (FGF-19 and C4) in predicting BAM; and
5. Whether the biomarkers can provide information on the nature of BAM.

The tertiary research objective is, for established cut-off values, to compare the sensitivity and specificity of the SeHCAT test with those of the 2 biomarkers in providing a positive indication of BAM using trial of treatment with BAS as the gold standard.

The research objectives can be translated into the following research questions:

1. Is SeHCAT an accurate test to provide a positive indication of BAM and what are the optimal cut-off thresholds?
2. Can different cut-off thresholds be established for the different clinical populations under study?
3. How do the cut-off thresholds currently in use for SeHCAT in UK centers compare with these optimal values?
4. Is SeHCAT an accurate test to grade the severity of BAM?
5. Does adherence to treatment with BAS differ for the different clinical populations studied?
6. Can biomarkers provide an accurate test for a positive indication of BAM?
7. Is the accuracy of biomarkers higher than that of SeHCAT in giving a positive indication of BAM?

## Methods

### Type of Study

The protocol describes a multicenter prospective study. Patients referred for suspected BAM will undergo a SeHCAT test and be tested for the biomarkers before undergoing treatment with BAS. Response to BAS treatment will provide a gold standard indication of a diagnosis of BAM. The diagnostic accuracy of the SeHCAT test and of the biomarkers will be assessed for the prediction of BAM.

### Setting

For a UK study, participating centers may be identified from the pool of 38 centers that took part in the recent KiTEC survey.

The proposed selection criteria for the centers are (1) a minimum workload of 30 SeHCAT patients per annum for the most recent calendar year for which data are available; (2) agreement to adopt a standardized SeHCAT test procedure; (3) laboratory capacity to collect, prepare, and deliver biological samples (blood and feces) for analysis at a remote site; and (4) formal commitment of a consultant gastroenterologist to participate in the study.

### Study Population

Participants in the study will be adults recruited from a population of patients attending a secondary care gastroenterology appointment, presenting with chronic diarrhea of unknown cause, in whom BAM is considered clinically possible. Patients will be included if they are suspected of having BAM Type 1 (following ileal resection, ileal disease including Crohn’s disease, or bypass of the terminal ileum), BAM Type 2 (primary idiopathic malabsorption), or BAM Type 3 (secondary to other conditions, including cholecystectomy, peptic ulcer surgery, chronic pancreatitis, and celiac disease). Potential participants will be screened for eligibility using a structured questionnaire and detailed clinical assessment.

### Exclusion Criteria

Potential participants may not enter the study if ANY of the following apply:

1. They are unable to provide informed consent;
2. They are on medication that could confound follow-up assessment;
3. They are pregnant or they are at risk of pregnancy and do not wish to take adequate precautions against pregnancy for the duration of the study;
4. They have any severe comorbidity condition with less than 12 months life expectancy;
5. They are unable or unwilling to undergo a SeHCAT procedure following the standard protocol and/or to provide blood and fecal samples; or
6. They are unwilling to undergo a course of treatment with BAS.

### Study Procedure and Outcome Measures

**Figure 1:**
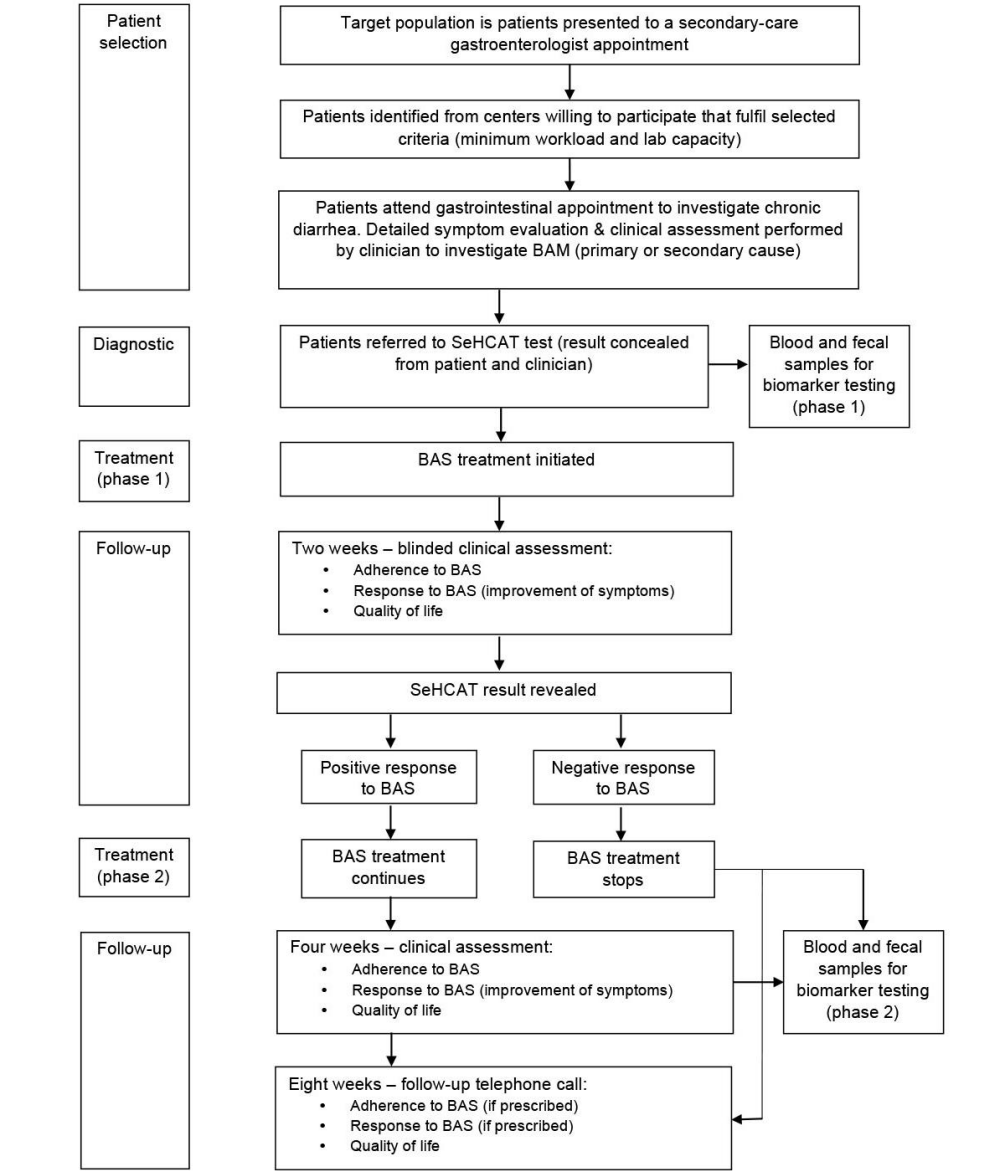
Study procedure.

A diagram illustrating the study procedure is shown in Figure 1. All patients attending a gastrointestinal appointment at a participating center and fulfilling the inclusion criteria will be invited to participate in the study. Individual patient consent will be requested at entry by the recruiting clinician. All patients who fulfill the inclusion criteria will be given detailed information both verbally and in the form of a patient information sheet. During the gastrointestinal appointment, patients will be referred by the clinicians for a SeHCAT test. The patients will be given detailed information of all relevant pretest and posttest dietary and medication requirements.

When the patient visits the hospital for the SeHCAT test, blood and fecal samples will be collected before the test using standard clinical procedures. The samples will be transferred to the local biochemistry laboratory at each participating site, where they will be handled by a trained biomedical scientist and prepared for transfer to the reference laboratory. The preparation procedure will involve taking a blood sample in a 5-mL tube containing no anticoagulants. This sample will be centrifuged in a standard bench-top centrifuge for 15 minutes at 3000 rpm, and the resulting serum separated from the cells and stored at –20°C before shipment to the central laboratory. A random fecal sample will be collected and stored at –20°C before shipment. The samples will be shipped on dry ice to the reference laboratory, where the serum samples will be analyzed for FGF-19 by enzyme-linked immunosorbent assay (ELISA), and for C4 and fractionated bile acids by liquid chromatography–mass spectrometry (LC-MS) using established techniques.

The SeHCAT test will be performed in the Nuclear Medicine Department of each participating center, following a standard protocol, under the supervision of an Administration of Radioactive Substances Advisory Committee (ARSAC) certificate holder [13]. A capsule containing radiolabeled SeHCAT will be administered to the patient orally according to a standard procedure and considering manufacturer’s recommendations. Patients will receive specific instructions regarding their food intake. After 3 hours, a scan will be performed to measure the baseline SeHCAT activity.

The patient will be requested to return to the Nuclear Medicine Department on day 7 to undergo a second scan to measure the retention of SeHCAT, thus completing the test. The SeHCAT test result will be concealed from the clinician and the patient until the 2-week follow-up appointment. On the visit to complete the SeHCAT test, BAS will be dispensed to allow the patient to commence treatment on the following day. BAS will be prescribed to all patients according to a standard protocol based on current clinical practice.

All patients will attend a follow-up appointment with the gastroenterologist 2 weeks after the initiation of treatment with BAS. A clinical assessment will be performed to capture the relevant outcome measures: the patient’s adherence to treatment, improvement of symptoms, and quality of life (QoL). The QoL assessment will be undertaken by a research nurse trained in qualitative methodology. The clinical assessment will determine whether there has been a positive or negative response to BAS treatment. A standardized definition will be agreed for the degree of improvement in symptoms required to represent a positive response to treatment. At the follow-up appointment, the SeHCAT test result will be revealed to the clinician and the patient. A positive response to treatment at 2 weeks with evidence of treatment adherence will be used as the gold standard that defines a diagnosis of BAM.

Patients with a negative response to treatment will stop BAS treatment promptly (irrespective of SeHCAT test result) and may be directed to other investigations. At this stage, blood and fecal samples will be collected from this group for assessment of biomarkers. Patients with a positive response to BAS treatment (irrespective of SeHCAT test result) will continue on BAS treatment for a further 2 weeks. For this group, a second follow-up appointment will be performed at 4 weeks to repeat the outcome assessment. At this second follow-up visit, blood and fecal samples will be collected for further biomarker testing.

At 8 weeks, all patients entered into the study will be contacted for a telephone follow-up appointment to collect final follow-up data. Any interventions other than those included in the study protocol will be recorded during the study period.

### Data Collection

Relevant clinical data regarding the care pathway, diagnostic tests, and interventions will be collected using electronic case report forms. Patient follow-up data will be collected using a questionnaire to be developed according to the Diagnostic Criteria for Functional Gastrointestinal Disorders (“the Rome criteria”) [14]. Quality of life measures will be captured using the standard EQ-5D tool [15].

All study data will be entered onto a database by qualified research staff and subjected to quality control checks. Patient-identifying details will not be included in any study data electronic files. After the closure of the study, the participating sites will maintain all source documents, study-related documents, and copies of the paper source documentation forms, data query, and amendment forms in compliance with local center policies. All source documents will be retained for a period of 10 years following the end of the study.

### Sample Size

Sample size was calculated based on the 95% confidence intervals of the sensitivity and specificity of the SeHCAT test result in predicting true BAM as defined by response to BAS treatment [16]. The prevalence of BAM in this population of patients attending gastroenterology appointments with chronic diarrhea is estimated to be approximately 50% [6]. Assuming a minimum value of 70% for either sensitivity or specificity [7], a sample of 600 patients (and therefore 300 true positives) would give an acceptable 95% confidence interval of 65% to 75%. Sensitivities or specificities closer to 1 will have narrower confidence intervals. If the assumption of 50% true positives is incorrect, an alternative scenario of 3:1 positives to negatives would give a 95% confidence interval of 62% to 77% around a specificity of 70%.

### Statistical Analysis

Results of the SeHCAT test (percentage retention per patient) will be presented using suitable descriptive methods, including frequencies, proportions, means, and standard deviations, as appropriate. Response to BAS treatment will be presented as a proportion of those who initiated the treatment. All descriptive statistics will be presented with 95% confidence intervals.

The diagnostic accuracy of the SeHCAT test to provide a positive indication of BAM compared with the gold standard of a positive response to BAS at 2 weeks of treatment adherence will be investigated using the receiver operating characteristic (ROC) curves approach to compare different choice of thresholds. Sensitivity, specificity, positive predictive value, negative predictive value, likelihood ratios, and area under the curve will be calculated and presented with 95% confidence intervals. Multivariable logistic regression models will be used to assess the association between presence of BAM and continuous SeHCAT retention levels after adjustment for confounders.

Similar methods will be used to assess the diagnostic accuracy of biomarkers in the prediction of BAM. The diagnostic accuracy of the selected biomarkers will be compared to that of the SeHCAT test using the McNemar test for paired proportions.

Where diagnosis of BAM is confirmed, the association between severity of BAM (as assessed clinically at baseline) and the continuous SeHCAT result will be investigated using appropriate multivariable regression models taking into account prognostic baseline variables as potential confounders.

The EQ-5D scores [15] will be derived using standard procedures and will be used to describe the QoL of different subgroups. The change in QoL for the SeHCAT-positive group who respond positively or negatively to BAS will be examined and compared using regression methods adjusting for prognostic variables including QoL at baseline.

All analyses will be 2-sided with 5% significance level and will be reported according to the international standards for the reporting of diagnostic accuracy studies [17].

### Ethics and Governance

Individual patient consent will be requested at entry by the recruiting clinician. All patients who fulfill the inclusion criteria will be given detailed information by a qualified researcher both verbally and in the form of a patient information sheet. Patients will be able to withdraw consent at any time during the study. Patients may also be withdrawn from the study for medical reasons.

Permission and approval for the proposed research will be requested using the relevant procedures for ethical review of studies of NHS patients in the United Kingdom. The protocol will be reviewed by an NHS Research Ethics Committee, overseen by the UK Health Research Authority. Local research and development approval will also be obtained from each participating center.

The chief investigator will be responsible for supervising the conduct of the research and for protecting the rights, safety, and welfare of the participants enrolled in the study. Principal investigators will be identified at each participating site to ensure that the research activities are conducted in an ethical manner and in accordance with UK regulations, institutional policies, and good clinical and research practice.

### Safety and Adverse Incidents

All adverse events will be recorded and acted on by a qualified member of staff (eg, a nurse under the supervision of the clinician). The SeHCAT test is standard practice and is a safe test that has been in use in the United Kingdom for more than 30 years. The SeHCAT radiolabeled synthetic bile acid is licensed and approved for use in the United Kingdom. The test involves exposure to a low dose of ionizing radiation. All procedures will comply with ARSAC regulations and patients will be given radiation protection instructions both verbally and in writing. The BAS drugs to be prescribed are licensed and used in standard practice, and will be used in full compliance with the licensed indication.

### Study Committees

A multidisciplinary Study Management Group will be responsible for running the study on a day-to-day basis and will include a manager, chief investigator, clinicians, and statisticians. A Study Steering Committee will take major decisions, such as changing the protocol, and will include members who are not involved with the running of the study. A Data and Safety Monitoring Committee will be established and will consist of at least 3 independent members, one of whom will be a clinical specialist and one a statistician. This committee will be responsible for assessing recruitment data and study conduct and the monitoring of adverse events. The research team will include external independent members, including a patient representative on the committees, to aid the team in making unbiased decisions free from financial, personal, or professional pressure.

## Results

Funding is currently being sought to carry out this proposed research.

## Discussion

### Strengths

The SeHCAT test for diagnosis of BAM has been in common use in UK nuclear medicine centers for more than 30 years. An evidence-based assessment of the accuracy of the SeHCAT test is overdue. This research is seen as a priority by the National Institute for Health and Care Excellence in the United Kingdom [18], who funded the recent prospective study of UK centers [6] and the development of this protocol.

Chronic diarrhea is a debilitating condition that is not only physically unpleasant to endure, but impacts on social engagement and quality of life. Improving diagnostic accuracy in the prediction of BAM should help lead to optimization of treatment for those patients whose diarrhea is linked to this condition and clinicians will be able to advise patients with greater confidence.

The recent UK review [4] and survey [6] suggest that the timing is currently optimal for conducting the proposed research. Up-to-date contextual baseline data are available and UK centers have recently engaged in the provision of data, with a database of contacts available for centers, all of which should improve the feasibility of conducting the study.

### Challenges

A positive response to BAS treatment is considered here as the gold standard for the diagnosis of BAM; a negative response will be coded as the patient not having BAM. However, this assumes that BAS is always effective in patients suffering from BAM. Without further research, it is impossible to assess whether this is a realistic claim. Similarly, it is possible that patients may have a placebo response (ie, an improvement in clinical symptoms even if they do not have underlying BAM). Longer-term follow-up may help to assuage these concerns.

Some forms of BAS treatment are unpleasant and poorly tolerated, including cholestyramine and colestipol, due to the texture and taste of the resin powder. This may reduce both the willingness of patients to take part in the study and adherence to treatment once started. Recruitment may also be affected by the fact that *all* patients will receive BAS regardless of whether SeHCAT indicates any likely benefit. However, in this regard, maintaining blindness to the SeHCAT result should help to retain participants once they have agreed to take part. These issues could be surmounted by using the drug colesevelam, which is available in tablet form and is well tolerated. However, colesevelam has not been assessed in randomized controlled trials for the treatment of BAM and is not currently licensed in the United Kingdom for this purpose [19].

Conducting a prospective multicenter study presents its own challenges, which include potential variation in procedures and in data quality between centers and the need for effective management of centers to ensure study timelines are achieved. To address these challenges, standardized protocols will be developed for the administration of the SeHCAT test and the laboratory assessment of biomarkers, and definitions will be agreed for all clinical assessments, including those required to assess patient eligibility and response to treatment. A consistent approach will be developed for communicating to patients the potential benefits as well as adverse effects of BAS treatment to encourage optimum treatment adherence. The study manager will be responsible for liaising with centers to ensure that agreed procedures are observed, that patients are recruited and followed up, and that data are complete and uploaded to the database in a timely fashion.

### Future Directions

The results from this study will need to be validated in other populations in the future. Populations with different dietary content, such as proportion of fiber, may react differently to the SeHCAT test [8]. Analysis of the variation in SeHCAT results between different centers will give an indication of the extent to which population-specific approaches may be required.

Depending on the results found for the 2 biomarkers considered, other biomarkers may be worth investigating as well as the potential for combining the SeHCAT and biomarker results.

## Abbreviations

C4: 7-alpha-hydroxy-4-cholesten-3-one
ARSAC: Administration of Radioactive Substances Advisory Committee
BAM: bile acid malabsorption
BAS: bile acid sequestrant
ELISA: enzyme-linked immunosorbent assay
FGF-19: fibroblast growth factor 19
KiTEC: King’s Technology Evaluation Centre
NIHR: National Institute for Health Research
QoL: quality of life
SeHCAT: tauroselcholic (selenium-75) acid
